# Single molecule studies reveal that p53 tetramers dynamically bind response elements containing one or two half sites

**DOI:** 10.1038/s41598-020-73234-6

**Published:** 2020-09-30

**Authors:** Elina Ly, Jennifer F. Kugel, James A. Goodrich

**Affiliations:** Department of Biochemistry, University of Colorado Boulder, 596 UCB, Boulder, CO 80309 USA

**Keywords:** Transcription factors, Single-molecule biophysics

## Abstract

The tumor suppressor protein p53 is critical for cell fate decisions, including apoptosis, senescence, and cell cycle arrest. p53 is a tetrameric transcription factor that binds DNA response elements to regulate transcription of target genes. p53 response elements consist of two decameric half-sites, and data suggest one p53 dimer in the tetramer binds to each half-site. Despite a broad literature describing p53 binding DNA, unanswered questions remain, due partly to the need for more quantitative and structural studies with full length protein. Here we describe a single molecule fluorescence system to visualize full length p53 tetramers binding DNA in real time. The data revealed a dynamic interaction in which tetrameric p53/DNA complexes assembled and disassembled without a dimer/DNA intermediate. On a wild type DNA containing two half sites, p53/DNA complexes existed in two kinetically distinct populations. p53 tetramers bound response elements containing only one half site to form a single population of complexes with reduced kinetic stability. Altering the spacing and helical phasing between two half sites affected both the population distribution of p53/DNA complexes and their kinetic stability. Our real time single molecule measurements of full length p53 tetramers binding DNA reveal the parameters that define the stability of p53/DNA complexes, and provide insight into the pathways by which those complexes assemble.

## Introduction

Transcriptional activators control when and in what cell type specific genes are expressed, a regulatory process that underpins all aspects of cell biology. p53 is a critical transcriptional activator that controls cell fate decisions including apoptosis, senescence, and cell cycle arrest in response to stresses such as DNA damage^1,2^. Because p53-dependent transcriptional pathways are activated in response to genotoxic stress, p53 is often referred to as the guardian of the genome^3,4^. Indeed, p53 mutations—often impairing its ability to bind to DNA response elements (REs)—result in carcinogenic phenotypes across a broad spectrum of cell types^5^, and p53 is one of the most commonly mutated genes in human cancers^6,7^. p53 is also important for regulating cellular metabolism in response to low levels of constitutive stress^2^. In all cases, the ability to activate transcription is key to how p53 functions, underscoring the importance of unraveling the molecular mechanisms by which it activates transcription.

To activate transcription, p53 must recognize and bind to p53 REs in DNA. The p53 RE is composed of two 10 bp half site sequences with 0–13 base pairs of spacing in between^8,9^. Each half site is defined by the consensus sequence RRRCWWGYYY, where R represents A or G, W represents A or T, and Y represents C or T^8,9^. A p53 RE can be located proximal to the core promoter of a gene it regulates, or many kilobases away within enhancer regions^10^. Genome-wide data sets interrogating p53 occupancy reveal hundreds to thousands of binding sites, depending on the cell type and conditions^11–13^. Current understanding of how p53 binds its RE has been informed from numerous biochemical and structural studies that describe unifying principles^14^; however, unanswered questions remain. For example, the majority of structural and biochemical studies describing p53/DNA interactions do not use the full length p53 protein. Therefore, the contributions of regions such as the N-terminal and C-terminal domains of p53 to parameters such as binding affinity, kinetic stability, and oligomeric state, are unclear. This is particularly important in light of more recent evidence showing that regions of p53 outside of the DNA binding domain can impact the interaction of p53 with DNA^15–20^.

**Figure 1:**
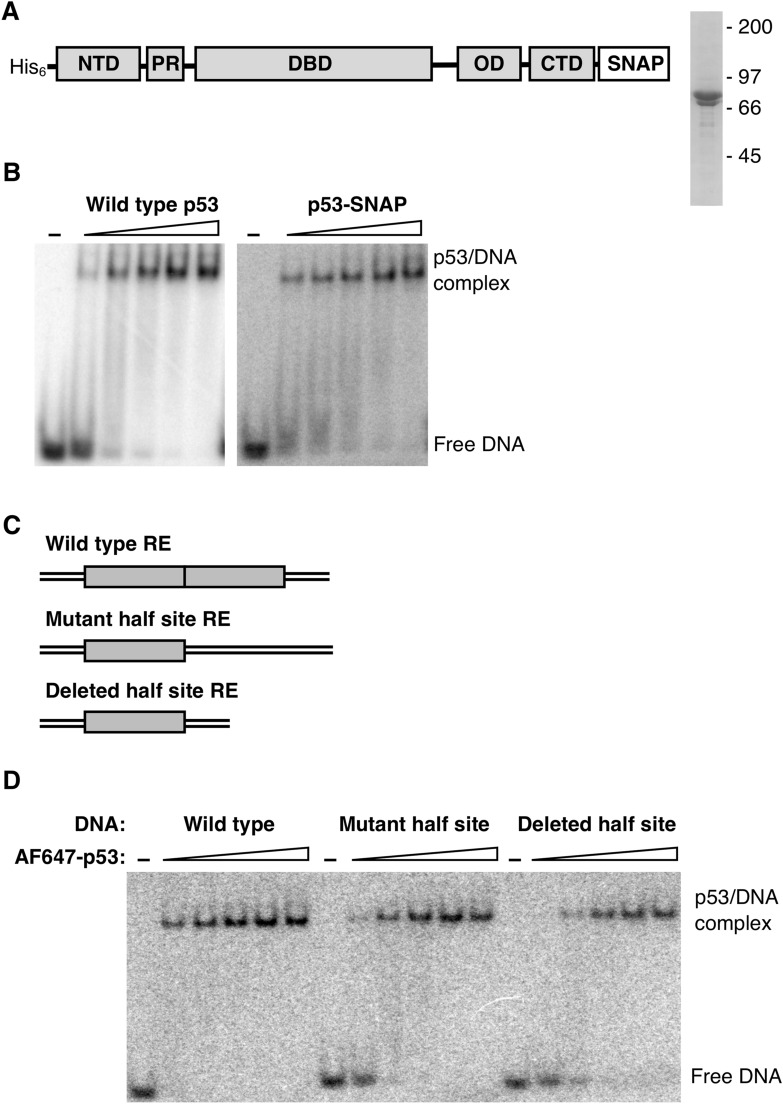
Full length p53 labeled with AF647 via a SNAP tag binds DNA with sub-nM affinity in EMSAs. (**A**) Schematic showing the domain structure of human full length p53 with the His tag used for purification and the SNAP tag used for fluorescent labeling. Also shown is the purified protein resolved by SDS-PAGE. The positions of the molecular weight markers are indicated; the less abundant band is protein lacking the His tag. An uncropped image of the gel is shown in Supplementary Fig. 7. The abbreviations are as follows: NTD, N-terminal domain; PR, proline-rich domain; DBD, DNA binding domain; OD, oligomerization domain; CTD, C-terminal domain. (**B**) The addition of a C-terminal SNAP tag to full length p53 does not appreciably alter DNA binding activity. The wild type p53 and p53-SNAP titrations were as follows (maximal tetramer concentrations): 0.025, 0.075, 0.25, 0.75, and 2.5 nM. In all experiments p53 concentrations are expressed as maximum tetrameric concentration, given the monomer concentrations added to the assays. Uncropped images of the gels are shown in Supplementary Fig. 7. (**C**) Schematics showing the half site composition of the p53 REs tested. The wild type 32 bp DNA is from the GADD45 promoter and consists of two 10 bp half sites (gray boxes) with no spacing between them. The mutant half site DNA was the same length (32 bp) and had one half site scrambled, whereas the deleted half site DNA had one half site removed and was therefore shorter (22 bp). (**D**) p53 binds DNA containing one or two half sites with high affinity and the same oligomeric state. The AF647-p53 titration was as follows (maximal tetramer concentration): 0.075, 0.25, 0.75, 2.5, and 7.5 nM. An uncropped image of the gel is shown in Supplementary Fig. 7.

The domain structure of p53 (Fig. 1A) consists of an acidic N-terminal region, a core DNA binding domain (DBD), an oligomerization domain (OD), and an unstructured basic C-terminal domain (CTD)^14^. The N-terminal region contains two unstructured transcriptional activation domains that interact with important co-regulatory complexes such as mediator, subunits of TFIID, and chromatin modifiers (p300 and GCN5, for example) to mediate transcriptional activation^21^. Recent work revealed that intramolecular interactions between the N-terminal region and the DBD can impact the affinity and specificity with which p53 binds DNA^19,20^. The DBD is largely responsible for recognizing and making contacts with p53 REs. It contains the highest frequency of cancer associated mutations, highlighting the importance of DNA binding to the function of p53^22^. The OD facilitates the formation of the p53 tetramer, commonly thought of as a dimer of dimers. Tetrameric p53 is considered the fully active DNA-bound form^23–25^; however, some data suggest that dimeric p53 can trigger specific transcriptional programs in cells^26,27^. The role of the basic unstructured CTD is more enigmatic, and the majority of biochemical studies of p53 DNA binding use versions of the protein lacking the CTD. Initially the CTD was thought to negatively regulate DNA binding, but more recent work has shown the CTD can stabilize p53 binding to REs as they diverge from consensus^15,16^, and also facilitate DNA sliding as p53 searches for its specific binding sites^17,18^.

Crystal structures of the isolated DBD bound to a single DNA half site revealed two DBD monomers bound per half site, with two such dimer/DNA complexes packing into tetramers, leading to the model that p53 binds DNA as a dimer of dimers^28^. The structure of the p53 DBD bound to a complete RE (i.e. two half sites) also revealed tetramers binding DNA (one dimer per half site) with extensive protein–protein contacts occurring at the interface between the two dimers^29^. To date high resolution structures of full length p53 bound to DNA have not been solved. Lower resolution EM studies with full length p53 suggest a p53 tetramer can bind a single half site, and moreover, that an octamer can assemble on the DNA if p53 tetramers bind both half sites present in an RE^30–32^.

To provide insight into the fundamental mechanisms by which p53 binds to DNA, we developed a single molecule assay to measure binding and dissociation of full length human p53 on DNA molecules in real time. We investigated binding to the p53 RE from the GADD45 promoter, as well as mutations of this RE that eliminated one of the consensus half sites or changed the spacing between half sites. We found that p53 tetramers dynamically bound to and dissociated from DNA, with many of these events occurring over minutes. Our data showed that tetrameric p53/DNA complexes did not assemble and disassemble via a dimer/DNA intermediate, and on the wild type RE, complexes were found in two kinetically distinct populations. We found that p53 tetramers could bind to DNAs containing only a single half site, as well as DNA with altered spacing between the half sites; however, the population distribution and kinetic stabilities of these complexes were different than complexes formed on the wild type DNA. Our studies report real time kinetic measurements of p53 tetramers binding to DNA and suggest new mechanisms by which this interaction occurs.

## Results

### Full-length p53 labeled with AF647 tightly binds DNA containing one or two half-sites

With the goal of visualizing p53 binding to DNA using single molecule microscopy, we generated full length human p53 with a C-terminal SNAP tag (p53-SNAP) for fluorescent labeling^33^. p53-SNAP was expressed in insect cells then purified via a His tag present on the N-terminus of the protein (Fig. 1A, right panel). We compared p53-SNAP and full length wild type p53 binding to the p53 RE from the GADD45 promoter using electrophoretic mobility shift assays (EMSAs) (Fig. 1B). The addition of the SNAP tag did not appreciably impact the DNA binding activity of p53. Next, the p53-SNAP protein was fluorescently labeled with an AlexaFluor647 SNAP dye substrate (AF647-p53). We determined there was near complete coupling of the AF647 dye to p53-SNAP using LC MS/MS. We also evaluated the extent of labeling using fluorescence and absorbance, which each indicated ~ 70% labeling efficiency. Therefore, we concluded that ~ 30% of the conjugated AF647 dyes were not photoactive, which is consistent with our previous observations^34^.

We tested the ability of AF647-p53 to bind the three DNA constructs diagrammed in Fig. 1C using EMSAs. The wild type DNA consisted of the RE from the human GADD45 promoter, containing two 10 bp half-sites (gray boxes) with no spacing between. The mutant half site construct had one half site sequence randomized and the other remained intact. The deleted half site construct was missing one half site, and therefore was 10 bp shorter in length compared to the other two constructs. These DNAs were ^32^P-labeled and incubated with increasing concentrations of AF647-p53, then free versus bound DNA was resolved in a native gel (Fig. 1D). The data indicate that full length p53 bound the wild type DNA with sub-nM affinity, assuming it bound as a tetramer, which is consistent with most models. When one half site was mutated or removed, the apparent affinity decreased slightly. The most striking observation from these data is that the bound DNA complexes on the three different DNAs migrated at identical positions in the gel. This indicates that p53 binds the wild type DNA and the single half site DNAs with the same oligomeric state, presumably a tetramer.

### A single molecule fluorescence microscopy assay to visualize DNA binding by p53 tetramers

**Figure 2:**
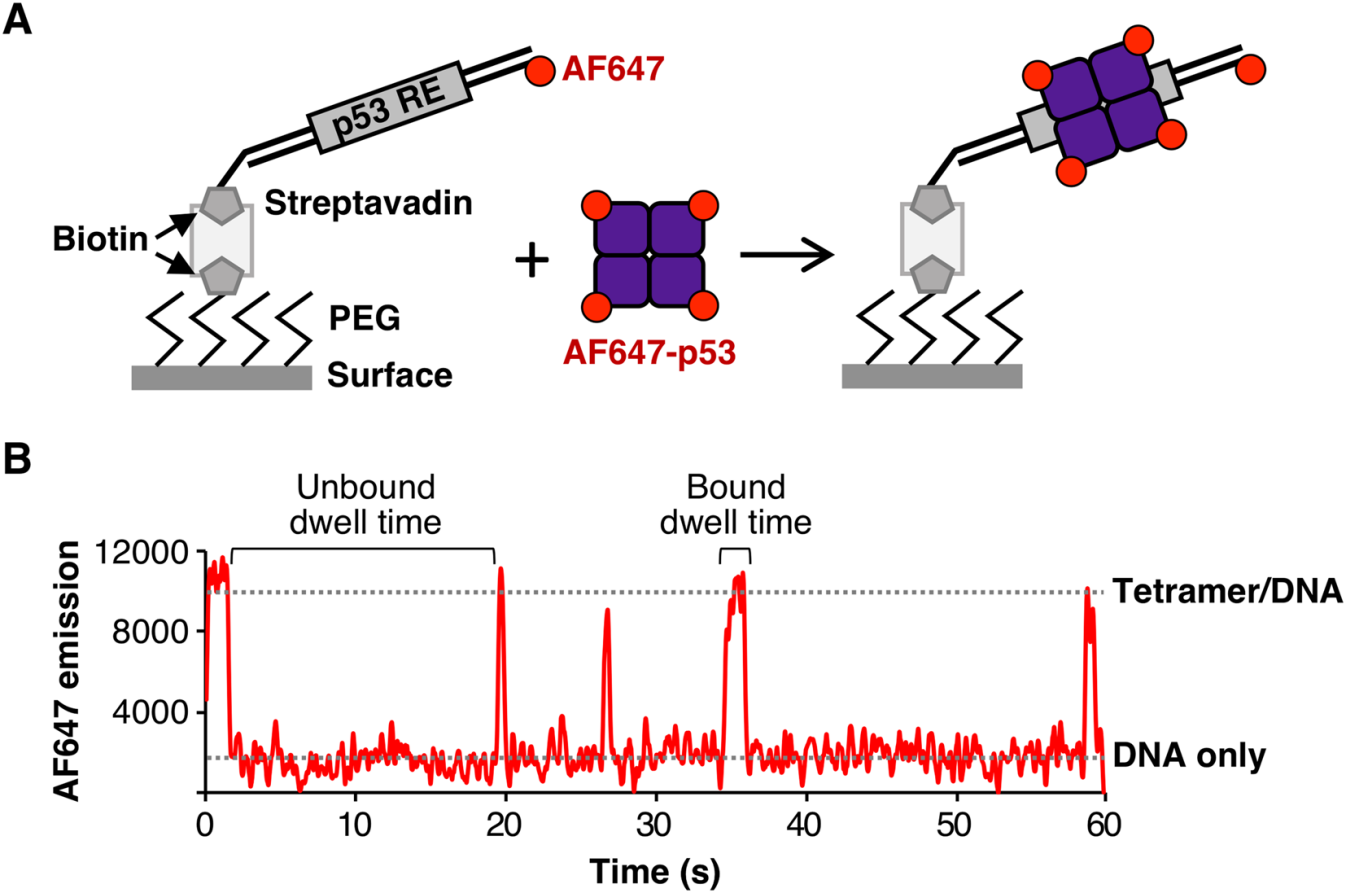
Single molecule fluorescence can be used to detect dynamic binding of tetrameric p53 to immobilized DNA in real time. (**A**) Illustration of the single molecule assay used to quantify tetrameric p53 binding to DNA. Red emission movies of biotinylated AF647 labeled DNA immobilized on the streptavidin derivatized surface were collected. AF647-p53 was flowed in and another red emission movie was collected over the same region (p53 + DNA). Co-localization of molecules in the DNA only data and the p53 + DNA data identified spot pairs from which emission intensity traces over time were extracted. These were used to calculate the base intensity value for a single dye on the DNA, and subsequently calculate the number of dyes (i.e. oligomeric state) of the p53 bound to DNA. (**B**) Representative AF647 emission data from a single DNA molecule with five tetrameric p53 binding/unbinding events, shown by the sharp increases and decreases in red intensity. Emission data were collected every 60 ms for 1000 frames. Representative regions used to obtain unbound and bound dwell times are indicated.

We next used AF647-p53 to establish a TIRF (total internal reflection fluorescence) microscopy system to quantify full length p53 binding to DNA in real time at the single molecule level. This would allow us to directly determine whether p53 binds single half site DNAs as a tetramer, and also measure how the kinetics of p53 binding is impacted by the number of half sites. As illustrated in Fig. 2A, biotinylated DNA constructs labeled with an AF647 fluorophore were immobilized on the surface of slides functionalized with streptavidin. To mark the positions of AF647-labeled DNA on the surface, images of red emission were obtained with a TIRF microscope (DNA movie). Subsequently, AF647-p53 was flowed into the slide chambers and incubated with the DNA for 10 min. A red emission movie was recorded over the same regions of the slide (DNA + p53 movie). Representative images of slide surfaces for DNA, DNA + p53, and p53 alone are shown in Supplementary Fig. 1. Spots of AF647 emission from the DNA movie were co-localized with spots of AF647 emission from the DNA + p53 movie. For each spot pair the fluorescence emission over time was followed to identify binding and dissociation events. Importantly, the AF647 on the DNA molecule provided a baseline intensity for a single red dye at that position on the surface. This baseline emission intensity was used to “count” the number of dye molecules associated with each AF647-p53 binding event, thereby providing information about its oligomeric state.

Figure 2B shows representative data for a single DNA molecule on which five p53 binding events occurred over 1 min, reflected by the five spikes in red emission intensity. The change in fluorescence emission during each of the binding and release events was ~ four-fold greater than the emission from the single red dye on the DNA, showing that each binding and dissociation event involved tetrameric p53. In principle, this approach could allow us to distinguish between dimer versus tetramer binding. However, since only ~ 70% of the AF647-p53 molecules contained photoactive dyes, a non-negligible fraction of dimer binding events could be due to partially labeled tetramers (i.e. tetramers containing only two photoactive fluorophores). Therefore, to be confident in our interpretations, we focused our studies only on tetrameric binding events that showed three or four active AF647 dyes binding and dissociating. Considering only tetrameric events also ensured that dissociation events were not due to photobleaching or photoblinking, which would not occur simultaneously for the three or four active AF647 dyes per protein. We evaluated the specificity of detecting p53/DNA binding interactions in this system using a rotated control (Supplementary Fig. 2). The percentage of DNA spots that had a co-localized AF647-p53 dropped from 37% to less than 3% when the AF647-p53 emission image was rotated 90º during spot pairing. This demonstrates that random co-localization in this system is quite low, with the potential for only 7% false positive colocalization.

### Single molecule studies reveal that p53 tetramers bind to DNAs containing either one or two half sites

We used our single molecule assay to determine whether p53 tetramers bind to DNAs containing one half site, as suggested by the EMSAs in Fig. 1C. Single molecule experiments were performed using AF647-p53 and the three DNAs depicted in Fig. 1B, but labeled with AF647 and immobilized on slide surfaces. We also tested a randomized DNA in which the entire p53 RE was scrambled to no longer resemble the consensus sequence. In all single molecule experiments we used 1 nM monomeric p53, which corresponds to a maximum tetrameric concentration of 0.25 nM. For each DNA we counted the number of dynamic tetrameric binding events over 1000 frames, taking data every 60 ms.

**Figure 3:**
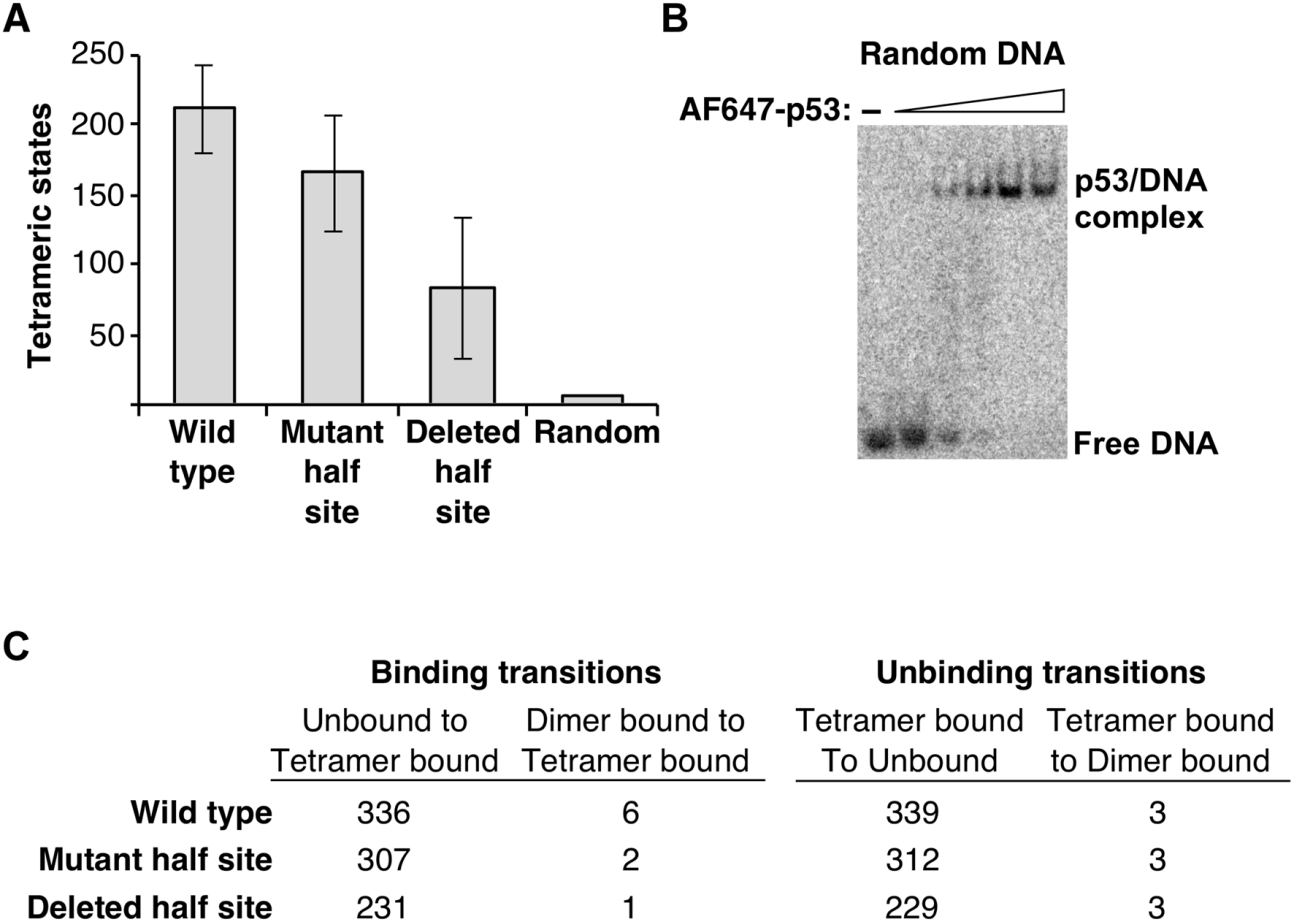
p53 specifically binds as a tetramer to DNA containing one or two half sites. (**A**) AF647-p53 binds as a tetramer to wild type DNA and DNAs with a mutated or a deleted half site. The bars represent the average number of dynamic tetrameric states observed from two experiments for the wild type and mutant half site DNAs, and three experiments for the deleted half site DNA. Data with the random DNA were obtained from one experiment over four slide regions. Error bars are the range of the measurements for the wild type and mutant half site DNAs and the standard deviation of the measurements obtained with the deleted half site DNA. The *p* values from an unpaired two-tailed *t* test are as follows: wild type versus mutant 0.48, wild type versus deleted 0.07, and mutant versus deleted 0.25. (**B**) p53 binds to DNA lacking a p53 binding site in EMSAs. The titration of AF647-p53 was as follows (maximal tetramer concentrations): 0.075, 0.25, 0.75, 2.5, and 7.5 nM. An uncropped image of the gel is shown in Supplementary Fig. 7. (**C**) Tetrameric p53 does not go through a dimer/DNA intermediate en route to binding or unbinding. Shown for each DNA are the number of binding and unbinding transitions across two experiments for a total of 8 slide regions.

Tetrameric AF647-p53 binding events were observed on the three DNAs with either one or two half sites. As described above, a tetrameric binding event shows three or four active AF647 dyes binding and dissociating. To illustrate this on the three DNAs, we histogrammed the ratio of bound p53 fluorescence to DNA fluorescence for hundreds of binding events on each of the three DNAs and found that the ratio for each peaked at 3 and 4 (Supplementary Fig. 3). As plotted in Fig. 3A, the average number of tetrameric binding events observed on the three DNAs were not statistically different from one another (*p* values for all pairwise comparisons were > 0.05 from a two-tailed unpaired *t* test). The average percentage of DNA spots that obtained a co-localized AF647-p53 in a region was 30 ± 4% for wild type DNA, 21.4 ± 0.3% for the mutant half site DNA, and 19 ± 4% for the deleted half site DNA. The binding events were specific for p53 half sites (be it one or two) because virtually no binding was observed on the random DNA. We also tested the random DNA for p53 binding using EMSAs. As shown in Fig. 3B, complexes were observed on the random DNA at low nM concentrations of AF647-p53 tetramers, which is consistent with prior ensemble experiments showing that p53 has nM affinity for random DNA^23,35^. It is likely the caging effect in the native gel trapped kinetically unstable p53/DNA complexes that were not detectable in the single molecule system due to rapid dissociation.

We also assessed the pathways by which p53 tetramer/DNA complexes formed and dissociated on each DNA molecule. In other words, we evaluated whether tetramer/DNA complexes formed or dissociated via a pathway involving a dimer/DNA intermediate. As shown in Fig. 3C, we counted the number of tetrameric binding transitions that showed unbound DNA going to a tetramer-bound state versus the number of dimer-bound states going to tetramer. The opposite transitions were counted for unbinding events. For all three DNA constructs, the vast majority of binding and dissociation events involved the simultaneous addition or loss of ~ 4 dyes. Fewer than 2% of the binding and dissociation events involved the gain or loss of two dye molecules en route to the final bound or unbound state. Sample traces of tetramer binding and unbinding events, as well as dimer-to-tetramer transitions are shown in Supplementary Fig. 4. We conclude that p53 binding events, regardless of one or two half sites, involve tetramers associating with and dissociating from the DNA molecules.

**Figure 4:**
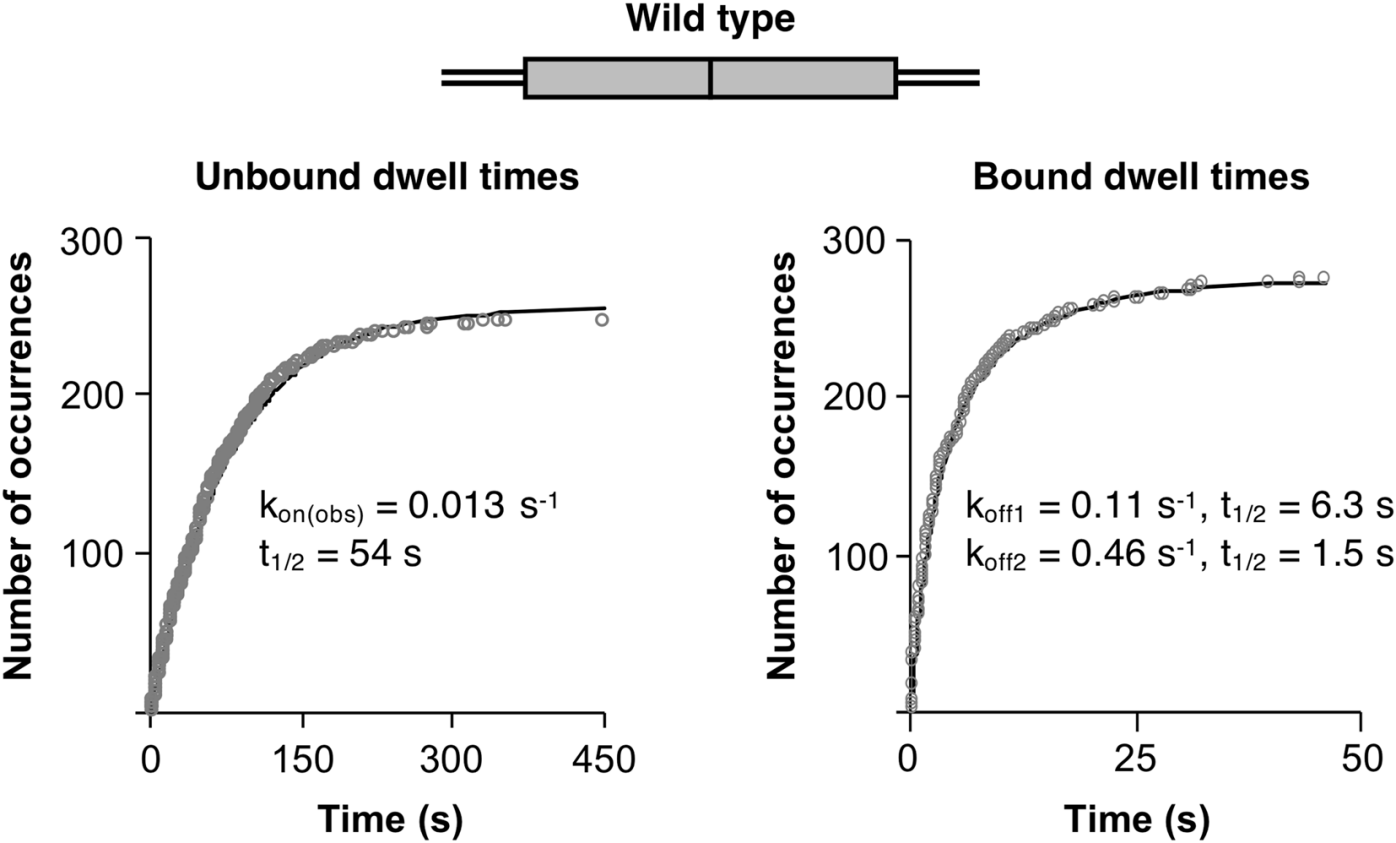
Under equilibrium conditions tetrameric p53 dynamically binds wild type DNA to form two populations of complexes with different kinetic stabilities. The left plot shows 492 unbound dwell times obtained from two experiments plotted as cumulative sums and fit with a single exponential equation. The 95% confidence interval for the curve fit is 0.012–0.013. The right plot shows 276 bound dwell times obtained from two different experiments plotted as cumulative sums and fit to a double exponential equation. The 95% confidence intervals for the curve fit are: k_off1_ 0.09–0.13, k_off2_ 0.42–0.52. The Y_max_ values of the double exponential curve fit reflect 49% and 51% of the population for the slow and fast dissociating complexes, respectively.

### p53 tetramers form two kinetically distinct complexes on the wild type RE

Given the observation that p53 tetramers bound all three DNAs containing at least one half site, we asked whether there were differences in the kinetics of binding or dissociation of tetramers to these three DNAs. We first measured the observed rate constant for association (k_on(obs)_) and the rate constant for dissociation (k_off_) on the wild type DNA. To obtain k_on(obs)_ we measured unbound dwell times (i.e. the time between two p53 tetramer binding events on a single DNA molecule, see Fig. 2B), and to obtain k_off_ we measured bound dwell times (i.e. the time each tetrameric p53 molecule remained bound to a DNA molecule). The dwell times from independent replicate experiments were combined, then plotted as cumulative sums and the data were fit to exponential equations to obtain rate constants and 95% confidence intervals of the curve fit (Fig. 4). The unbound dwell times (left plot) yielded a k_on(obs)_ of 0.013 s^−1^ on the wild type DNA, corresponding to tetrameric p53/wild type DNA complexes assembling with a half-time of 54 s (Table 1). The forward rate constant is presented as an observed first order rate constant because the measurement was made at a single concentration; we calculated a second order rate constant of 5.2 × 10^7^ M^−1^ s^−1^ using the maximal concentration of tetrameric p53 in the slide chamber.

**Table 1:** A summary of the kinetic constants for p53 tetramers binding to different DNAs under equilibrium conditions. nd is not determined.

DNA	On rate constants				Off rate constants			
	k_on(obs)_ (s^−1^)^a^	95% CI^b^	t_1/2_ (s)^c^	Tau (s)^d^	k_off_ (s^−1^)^a^	95% CI^b^	t_1/2_ (s)^c^	Tau (s)^d^
Wild type	0.013	0.012–0.013	54	78	0.11	0.09–0.13	6.3	9.1
					0.46	0.42–0.52	1.5	2.2
Mutant half site	0.0188	0.0185–0.0190	37	53	1.0	0.99–1.1	0.68	0.97
Deleted half site	nd				0.97	0.93–1.02	0.71	1.03
+ 5 bp insert	nd				0.12	0.03–0.24	5.8	8.4
					0.9	0.8–1.0	0.8	1.0
+ 10 bp insert	nd				0.48	0.47–0.49	1.4	2.1

^a^Data from two to four independent replicates were combined and fit with an exponential equation to obtain the rate constants shown.

^b^95% CI is the confidence interval of the rate constant obtained from the curve fit.

^c^Half-times were calculated using the equation for a first-order reaction t_1/2_ = ln2/k.

^d^Tau was calculated using 1/k.

When the cumulative sums plot of the bound dwell times was fit to obtain the rate constant for dissociation (k_off_) we observed that the data did not fit well to a single exponential equation (Supplementary Fig. 5A). These data were better fit by a double exponential equation, reflecting two kinetic populations of dissociation events with rate constants of 0.11 s^−1^ and 0.46 s^−1^ (Fig. 4, right plot), corresponding to half times of dissociation of 6.3 s and 1.5 s, respectively (Table 1). A plot of the residuals of the double exponential curve fit is in Supplementary Fig. 5B. The distribution of tetrameric p53/DNA complexes in each kinetic population was roughly equal with ~ 49% of complexes in the more stable population and ~ 51% in the less stable one. We calculated an apparent K_D_ for binding wild type DNA of ≤ 2 nM and ≤ 9 nM, for the more and less stable complexes, respectively; our calculations were made assuming all p53 in the reaction was tetrameric, hence the calculated K_D_ values are upper limits.

**Figure 5:**
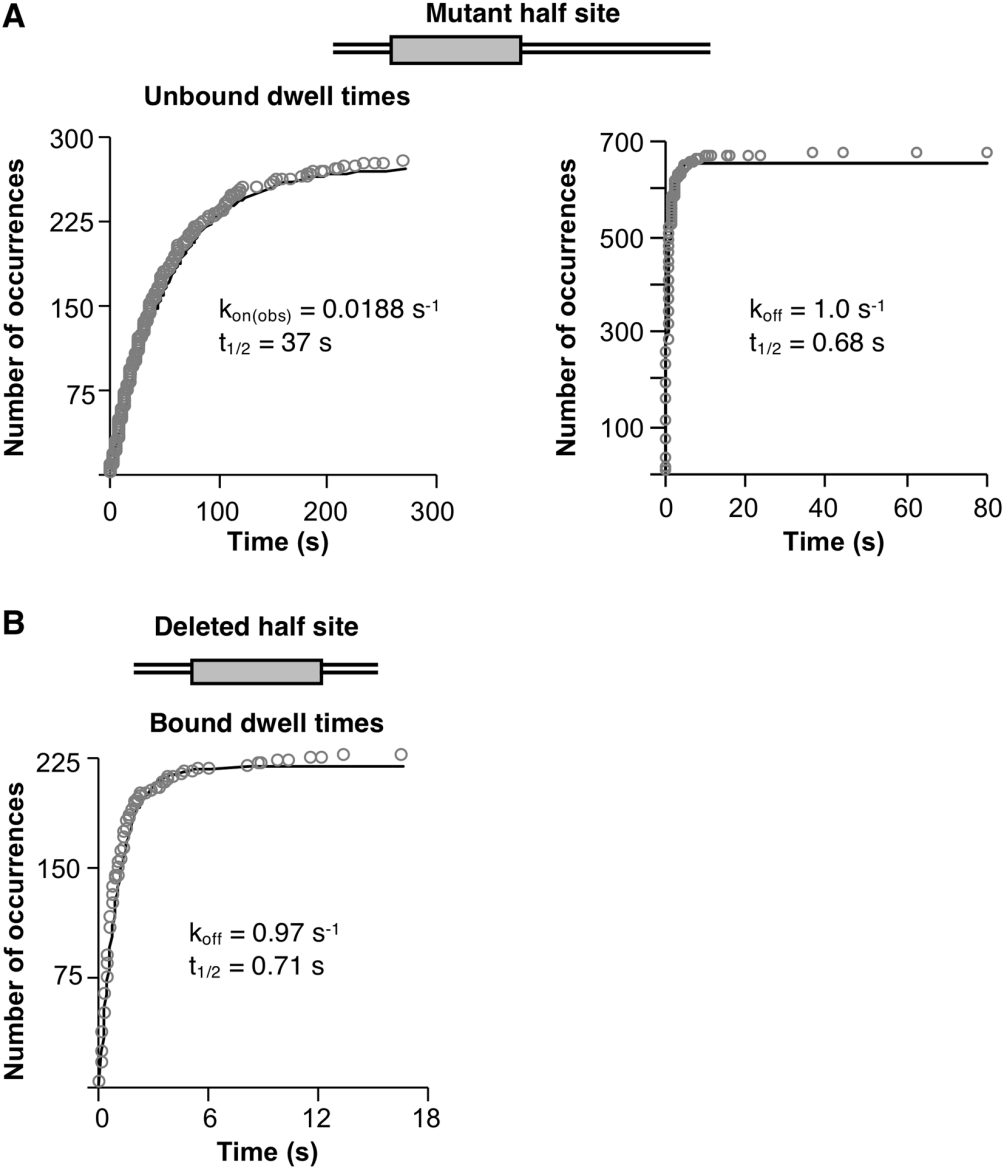
Eliminating one of the DNA half sites reduces the kinetic stability of p53/DNA complexes and results in dissociation characterized by a single rate constant. (**A**) Mutating one half site in the DNA does not change the rate of p53 binding, but increases the rate of complex dissociation. Cumulative sums plots of unbound (left plot) and bound (right plot) tetramer dwell times combined from four independent replicate experiments were fit to a single exponential equation. The plots shown include 276 unbound dwell times and 675 bound dwell times. The 95% confidence intervals for the curve fits are: k_on(obs)_ 0.0185–0.0190, k_off_ 0.99–1.1. (**B**) Tetrameric p53 binds to DNA containing a single half site with reduced kinetic stability. Bound tetramer dwell times from three experiments were combined and fit to a single exponential to obtain the k_off_ and a 95% confidence interval of 0.93–1.02. The cumulative sums plot shown includes 225 bound dwell times.

### p53 tetramers bind DNAs containing only one half site to form a single population of complexes with reduced kinetic stability

We next measured the unbound and bound dwell times for tetrameric p53 on the DNA with a mutant half site. As shown in Fig. 5A (left plot), fitting the unbound dwell times to a single exponential resulted in a k_on(obs)_ of 0.0188 s^−1^ (calculated second order rate constant of 7.5 × 10^7^ M^−1^ s^−1^). This is only slightly larger than the k_on(obs)_ measured on the wild type DNA; hence, mutating one of the half sites did not strongly affect the rate of association of p53 tetramers with the DNA. Interestingly, on the mutant half site DNA the bound dwell times fit well with a single exponential equation with a k_off_ of 1.0 s^−1^, corresponding to a half-time of 0.68 s (Fig. 5A, right plot). Therefore, mutating one of the half sites resulted in a single population of tetrameric p53/DNA complexes that is either two- or ten-fold less stable than complexes containing tetramers bound to wild type DNA with two half sites. The faster off rate on the mutant half site DNA results in lower binding affinity, with a calculated apparent K_D_ of ≤ 13 nM.

We asked if a similar reduction in the kinetic stability of p53/DNA complexes was observed on the DNA with one half site deleted. Indeed, the k_off_ on this DNA was 0.97 s^−1^ (Fig. 5B), which is similar to that measured on the mutant half site DNA. Together our data support the model that p53 tetramers bind different DNAs with the same frequency as long as at least one half site is present; however, the kinetic stability of tetrameric p53/DNA complexes is greater on the DNA containing two half sites (i.e. a full p53 RE). Moreover, tetrameric p53/DNA complexes assembled on two half sites are heterogeneous, consisting of two populations that have different kinetic stabilities.

### Increasing the spacing between half sites impacts the population distribution of p53/DNA complexes and alters the kinetic stability of bound tetramers

**Figure 6:**
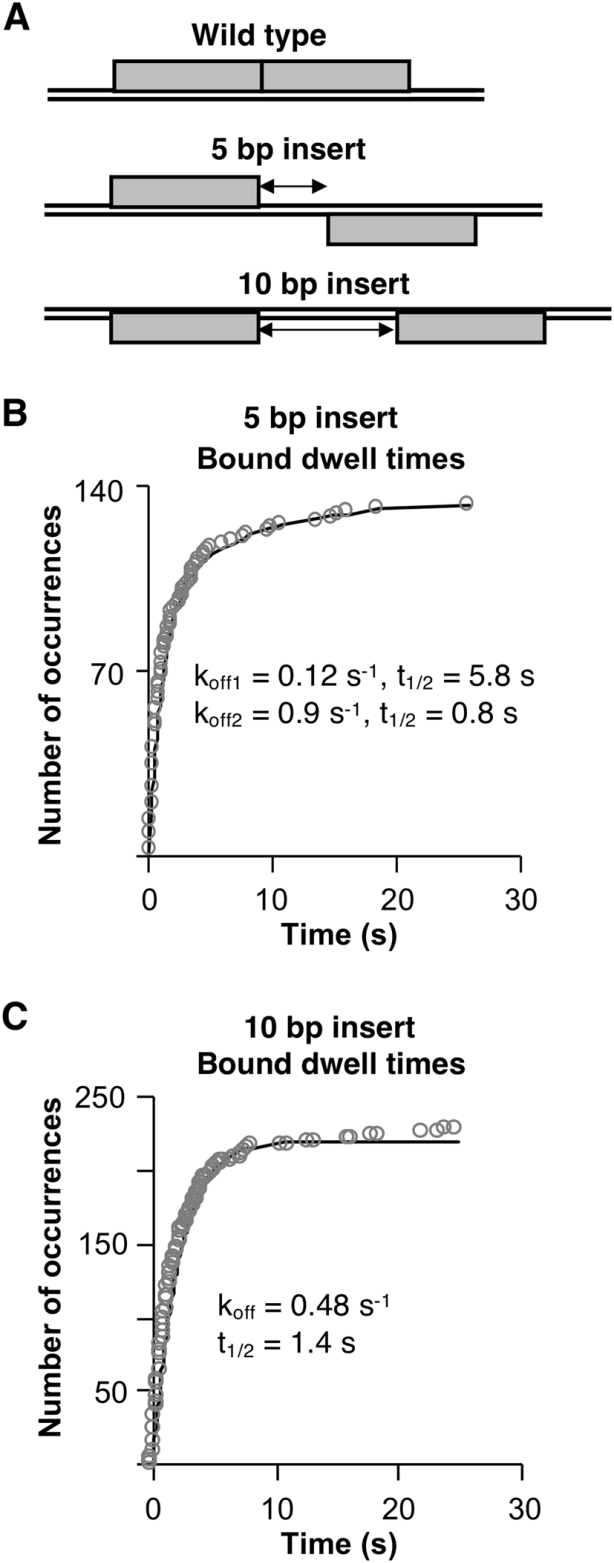
Changing the helical phasing between the half sites impacts the kinetic stability and population distribution of p53/DNA complexes. (**A**) Schematics depicting that in the wild type DNA the half sites are centered on the same face of the DNA helix, insertion of 5 bp between the half sites causes them to be centered on opposite faces of the DNA helix, and insertion of 10 bp results in the half sites being centered on the same face of the DNA helix. (**B**) Introducing a 5 bp spacer between the two half sites decreases the kinetic stability of one population of p53/DNA complexes. The plot shows bound dwell times from 133 states plotted as a cumulative sums and fit to a double exponential. The 95% confidence intervals for the curve fits are: k_off1_ 0.03–0.24, k_off2_ 0.8–1.0. The Y_max_ values of the curve fit reflect a 27% and 73% distribution between the slow and fast populations, respectively. (**C**) Increasing the spacing to 10 bp eliminates the more stable population of p53/DNA complexes and alters the kinetic stability of the other population. Shown is a cumulative sums plot of tetramer bound dwell times obtained from replicate experiments. 229 states were fit to a single exponential to obtain the k_off_ and a 95% confidence interval of 0.47–0.49.

p53 REs can have variable spacing between the two half-sites, ranging from 0–13 bp^8,9^. Hence the helical phasing between the two half sites could differ, which has the potential to impact the binding of p53 to DNA or the kinetic stability of complexes. To test this we designed DNA constructs containing either a 5 bp or 10 bp spacer between the two half sites. As illustrated in Fig. 6A, in the wild type DNA with zero spacing between the two half sites, the centers of the two half sites face the same side of the DNA helix. Adding a 5 bp spacer causes the centers of the two half sites to be on opposite faces of the DNA helix, whereas adding a 10 bp spacer restores the helical phasing such that the two half sites are centered on the same face of the DNA helix.

We measured the bound dwell times for tetrameric AF647-p53 on the 5 bp insert DNA. Similar to the wild type DNA, the data were best fit with a double exponential equation, giving two rate constants for dissociation of 0.12 s^−1^ and 0.9 s^−1^ (Fig. 6B). The residuals plots for single and double exponential fits of the data are shown in Supplementary Fig. 6. Although this comparison shows that the data are indeed best fit by a double exponential equation reflecting two kinetic populations, the 95% confidence interval (0.03–0.24) of the smaller rate constant (0.12 s^−1^) reflects substantial uncertainty in the value (Table 1). Therefore, we trust there are two kinetic populations of dissociating tetrameric p53/DNA complexes on the 5 bp insert DNA; however, we are not confident in the precise rate with which the more stable population dissociates. The less stable population (i.e. the larger rate constant) reflects a two-fold faster rate of dissociation compared to the less stable population on wild type DNA. Hence, centering the two half sites on opposite faces of the helix reduced the kinetic stability of p53/DNA complexes, but to a lesser extent compared to mutating or removing a half site.

We then tested how restoring the helical phasing between the two half sites would change the kinetic stability of tetrameric p53/DNA complexes, using the 10 bp insert DNA. The cumulative sums plot of the bound dwell times fit well with a single exponential equation, giving a k_off_ of 0.48 s^−1^ and a half-time of dissociation of 1.4 s (Fig. 6C). Hence complexes formed on the 10 bp insert DNA show a single kinetic population for dissociation that is similar to the less stable population that forms on wild type DNA. Therefore keeping the half sites centered on the same face of the DNA helix, but moving them apart results in loss of the more stable population of bound p53 tetramers. Together our data show that increasing the spacing between half sites differentially changes the kinetic stability and population distribution of bound tetramers, depending on whether the half sites are centered on the opposite or the same side of the DNA helix.

## Discussion

Crucial to the ability of p53 to induce transcription of genes is its recognition and binding of REs. To understand mechanisms by which DNA binding occurs we developed a single molecule fluorescence assay to quantify full length tetrameric p53 binding to DNA in vitro. We found that p53 tetramers bind dynamically and with high affinity to the p53 RE from the GADD45 promoter. Interestingly, our kinetic data with the GADD45 p53 RE indicate that two different forms of p53/DNA complexes exist that dissociate with rates that are four-fold different. When one of the two half sites was removed (via mutation or deletion), p53 tetramers still bound, and with the same rate of association. However, removal of one half site caused the p53/DNA complexes to dissociate as a single population with decreased kinetic stability. Increasing the spacing between half sites also affected the kinetic stability of p53/DNA complexes as well as the number of kinetically distinct complexes detected. Our real time kinetic measurements of tetrameric p53 binding to different DNAs reveal the parameters that define the stability p53/DNA complexes, and provide insight into the pathways by which those complexes assemble.

Our single molecule data provide insight into mechanisms by which p53 tetramers bind DNAs containing one or two half sites, as well as DNAs with altered spacing between half sites. With respect to the formation of p53/DNA complexes, the rates of association of p53 tetramers with wild type DNA and single half site DNA were indistinguishable, and nearly all binding events occurred in a single step (i.e. without a detectable dimer intermediate). These observations are consistent with a model in which p53 tetramers form in solution and then bind DNA. Given the concentration of p53 flowed into the slide chambers (1 nM monomeric) and the published K_D_ for tetramerization^36^, it is possible that the majority the AF647-p53 introduced into the chamber was not tetrameric. In which case, the rate of association of p53 with DNA could be set by the rate of formation of p53 tetramers, and once formed, the tetramers rapidly associate with the DNA. An alternative model is that two dimers bind sequentially to DNA at a rate faster than we can resolve; however, we believe this model is less likely. Presuming the second binding event would be diffusion limited, it would occur over several seconds at the p53 concentration used, which we would readily detect in our assays. With respect to the dissociation of p53/DNA complexes nearly all tetramer p53/DNA complexes dissociated without a detectable dimer/DNA intermediate, regardless of DNA construct (Fig. 3C). It is, however, formally possible that dimers dissociate sequentially at a rate faster than is detectable in our assays.

In the case of the wild type DNA containing two half sites, the binding of a p53 tetramer to both half sites on a DNA could occur simultaneously, or the initial recognition and binding could be driven by interactions between one dimer interface and a single half site. In the latter case, the initial interaction could be followed by a conformational change that allows the second dimer to interact with the other nearby half site, causing stabilization of the p53 tetramer/DNA complexes. Previous studies have suggested a two-step "induced fit" mechanism of binding in which initial DNA binding by p53 is followed by a conformational switch that helps control the dissociation rate^35,37,38^. Conformational transitions are not directly detectable in our single molecule assays; however, the following three observations are consistent with a stepwise association model for p53 tetramers binding a DNA with two half sites. First, our observation that the presence of two half sites stabilized the tetramer/DNA complex is consistent with this model. Second, on the wild type DNA there were two kinetic populations that had different rates of dissociation. It is possible that the more stable population reflects the dissociation of p53/DNA complexes in which each dimer interface is in contact with a half site, and the less stable population reflects complexes in which only one of the dimer interfaces is in contact with a half site at the time of dissociation. Third, p53 tetramers bound to DNAs containing a single half site behaved as a single population, dissociating with a rate that was within ~ two-fold of the least stable population dissociating from wild type DNA. Taken together, our data are consistent with a model in which p53 tetramers can bind DNAs containing two half sites in two modes—single half site binding and dual half site binding—with the possibility that the former is a precursor on the way to the formation of the latter.

It is possible that p53 tetramers bound to a single half site are structurally different from tetramers bound to two half sites. There are no high resolution structures of full length p53 bound to DNA, likely due to the unstructured N- and C-terminal regions interfering with structure determination. Because these domains impact the ability of p53 to bind DNA^15,19,20,39^, it reasonable to predict these regions could impact the structure of p53/DNA complexes. Interestingly, low resolution EM structures with full length p53 suggest two p53 tetramers can simultaneously bind each half site present in a full RE to form an octameric p53 oligomeric state^30–32^. Our finding that full length p53 binds DNA containing only one half site as a tetramer is consistent with these EM studies. We occasionally observed DNAs with higher order p53 oligomers in single molecule experiments (i.e. greater than four dyes bound to DNA); however, the number of these events was a very small fraction of the total binding events and were not considered in our analysis. Hence, we did not obtain strong support for the formation of octameric p53/DNA complexes in our experiments; however, it is possible that higher oligomeric complexes would be observed at concentrations of p53 much higher than that used in our studies.

Our single molecule experiments revealed that p53 binds and releases from DNA dynamically. This is consistent with mounting evidence that transcription factor/DNA associations are dynamic in mammalian cells^40–43^. For example, single molecule live cell tracking experiments using fluorescently labeled full length p53 reported average residence times of 3.1–3.5 s^44,45^, which is similar to the residence time for p53/wild type DNA that we measured in vitro.

We found that altering the spacing between DNA half sites changed the population distribution and kinetic stability of p53/DNA complexes. When 5 bp was added between the half sites there were still two kinetically distinct populations of p53/DNA complexes. Moreover, the least stable population on the 5 bp insert DNA dissociated faster than the least stable population on wild type DNA. The two half sites on the 5 bp insert DNA are centered on opposite faces of the DNA helix. If tetrameric p53 does utilize both half sites when binding this DNA, it would likely require deformation of the DNA (e.g. bending and/or twisting). Experimental and molecular dynamics data have suggested that p53 binding can induce DNA bending and/or twisting^46–50^. It is also possible that the two populations of complexes observed on the 5 bp insert DNA represent tetrameric p53 bound to one or the other of the two half sites, which are not identical. When 10 bp was added between the half sites, which retained the half sites being centered on the same face of the DNA helix, we observed only one population of p53/DNA complexes that dissociated with a rate constant identical to that measured for the least stable population on wild type DNA, and within twofold of those measured on DNAs with only a single half site. This is consistent with a model in which only a single half site on the 10 bp insert DNA is occupied during any binding event. Moreover, a prior study found that tetrameric p53 could bind to DNAs with longer spacing between half sites in a "hemispecific" mode, with one p53 dimer bound to a half site and the other dimer bound to the spacer DNA^51^. It is possible this mode of binding results in a kinetic stability that is twofold greater than binding a single half site. Understanding the precise relationship between the spacing between half sites, the kinetic stability of tetrameric p53/DNA complexes, and the conformation of DNA will require future experiments.

Although p53 is a widely studied protein, elucidating the mechanism by which it binds DNA is challenging due to the difficulty of studying the full length protein. Here we describe quantitative studies of full length p53 tetramers binding DNA in real time at the single molecule level. Our data provide important fundamental measurements, and insight into mechanisms by which this complex forms.

## Methods

### Cloning and expression of p53-SNAP protein

We created a fusion construct containing an N-terminal histidine tag, human full length p53, an 8-amino acid linker (GSSGGSSG), and a C-terminal SNAP tag. The p53-SNAP used in the experiment shown in Fig. 1A had the amino acid sequence KL prior to the linker. We used PCR with plasmids containing p53 or SNAP sequences and inserted isolated DNA fragments into the pACEBac1 baculovirus transfer vector via EcoRI and XbaI restriction sites. The p53-SNAP pACEBac1 plasmid was transformed into DH10EmBacY cells (Geneva Biotech) and cells were plated on agar plates for blue-white screening (50 µg/mL kanamycin, 10 µg/mL tetracycline, 7 µg/mL gentamycin, 100 µg/mL X-Gal, 40 µg/mL IPTG). A white colony, reflecting the correct transposition of the p53-SNAP insert into the bacmid DNA present within DH10EmBacY cells, was used to inoculate an overnight culture. Bacmid DNA, prone to fragmentation, was prepared from the culture using the following protocol. The cells were pelleted, then resuspended in 330 µL of solution I (15 mM Tris pH 7.9, 10 mM EDTA pH 8, 100 µg/mL RNase A, filter sterilized), followed by addition of 330 µL of solution II (0.2 M NaOH, 1% SDS, filter sterilized). The sample was inverted once to mix and incubated 5 min at room temperature, then 460 µL of 3 M potassium acetate (pH 5.5, autoclaved) was added, gently mixed by inverting, and incubated 5 min on ice. The sample was then centrifuged, the supernatant was transferred to a new tube, and the sample was subjected to a second centrifugation. The supernatant was transferred to a tube containing 900 µL of isopropanol and gently mixed by inverting. After a 15 min incubation on ice, the sample was centrifuged and the supernatant was removed. The pellet was washed with 70% ethanol then air dried for 5 min at room temperature. The pellet was solubilized in TE buffer (10 mM Tris pH 7.9, 1 mM EDTA pH 8, filter sterilized), avoiding mechanical resuspension. The DNA was filtered using a 0.22 µm Spin-X column (Fisher Scientific). Bacmid DNA was sent to the Protein Production/MoAB/Tissue Culture Shared Resource facility at the University of Colorado Anschutz Medical Campus to produce the baculovirus viral stock for infection of SF-9 insect cells to express p53-SNAP. Insect cell pellets were screened for protein expression using western blots against p53, the SNAP tag, and the His tag.

### Purification and fluorescent labeling of p53-SNAP

An SF-9 insect cell pellet expressing full length p53-SNAP was thawed on ice and resuspended in lysis buffer B (50 mM Tris pH 7.5, 150 mM NaCl, 10% glycerol, 0.5% NP-40, 2 mM DTT, 1 mM PMSF, 1 µg/mL Pepstatin A, 1 × EDTA-free Protease Inhibitor (Roche), 1 × PhosSTOP (Roche)) by vortexing then incubating on ice for 15 min before centrifugation. The supernatant was loaded onto a HisPur Ni–NTA resin (Thermo Scientific) column that was pre-equilibrated with lysis buffer B. The column was then washed sequentially with lysis buffer B, wash buffer A [50 mM Tris pH 7.5, 500 mM NaCl, 5 mM MgCl_2_, 10% glycerol, 2 mM DTT, 1 mM PMSF, 1 µg/mL Pepstatin A, 1 × EDTA-free Protease Inhibitor cocktail (Roche), 1 × PhosSTOP (Roche)], and wash buffer A containing 50 mM imidazole. p53-SNAP was eluted with wash buffer A containing 300 mM imidazole. Fractions were analyzed via SDS-PAGE, and the fractions containing the most p53-SNAP were pooled and dialyzed overnight in 50 mM Tris pH 7.9, 150 mM NaCl, 5 mM MgCl_2_, 10% glycerol, and 1 mM DTT. The purified protein was evaluated by western blots with antibodies against p53, the SNAP tag, and the His tag to confirm the identities of bands on SDS gels.

For the protein labeling reaction, 100 µL of p53-SNAP (diluted to a concentration less than 10 µM) was incubated with 2 µL of 1 mM SNAP-Surface Alexa Fluor647 dye (New England Biolabs) for 2 h at room temperature in the dark. Excess free dye was removed using Zeba Spin Desalting columns (7 K MWCO, 0.5 mL, Thermo Scientific). A coomassie stained SDS-PAGE gel with a BSA standard curve was used to calculate the total concentration of p53-SNAP. The concentration of AF647-p53 was evaluated by comparison to a standard curve of AF647-SNAP substrate and a standard curve of an AF647-labeled oligonucleotide using a Typhoon 9500 Imager. The absorbance at 652 nm and the molar absorptivity of AF647 was also used to calculate the concentration of AF647 in the labeled p53. In addition, LC–MS/MS was used to measure the amount of the 16-amino acid peptide (TALSGNPVPILIPCHR) containing the site of AF647 dye attachment in trypsin digested samples of unlabeled p53-SNAP and AF647-p53. The peptide was not detectable in the AF647-p53 sample, but readily detectable in the p53-SNAP sample, indicating complete conjugation to the dye.

### Oligonucleotides

The sequences of the DNA constructs used in binding assays were as follows (ordered HPLC purified from Integrated DNA Technologies): wild type reverse oligo, 5′CGACGCCAGCATGCTTAGACATGTTCGCTCTA3′ and wild type forward oligo, 5′TAGAGCGAACATGTCTAAGCATGCTGGCGTCG3′; mutant half site reverse oligo, 5′GACGCCGCCTTTGAAAGACATGTTCGCTCTA3′ and mutant half site forward oligo, 5′TAGAGCGAACATGTCTTTCAAAGGCGGCGTCG3′; deleted half site reverse oligo, 5′CGACGCAGACATGTTCGCTCTA3′ and deleted half site forward oligo, 5′TAGAGCGAACATGTCTGCGTCG3′; random DNA reverse oligo, 5′TATTCGTGTCAGCCCCTACCCGATTAGGAACG3′ and random DNA forward oligo, 5′CGTTCCTAATCGGGTAGGGGCTGACACGAATA3′; 5 bp insert reverse oligo, 5′CGACGCCAGCATGCTTTCCACAGACATGTTCGCTCTA3′ and 5 bp insert forward oligo, 5′TAGAGCGAACATGTCTGTGGAAAGCATGCTGGCGTCG3′; 10 bp insert reverse oligo 5′CGACGCCAGCATGCTTATTAATTCATAGACATGTTCGCTCTA3′ and 10 bp insert forward oligo 5′TAGAGCGAACATGTCTATGAATTAATAAGCATGCTGGCGTCG3′. For single molecule experiments, all reverse oligos contained a AF647 dye on the 5′ end and all forward oligos contained a 5′ biotin attached to a 24 nt single stranded linker: 5′CGCGTTCATGGTAGAGTCGTGGAC3′. For EMSAs, all reverse oligos were radiolabeled on the 5′end using T4 polynucleotide kinase and γ^32^P-ATP.

Double stranded DNAs were generated by annealing the forward and reverse oligos by heating to 95 °C for 10 min and slowly cooling to room temperature in the temp block on the bench top. For single molecule experiments, annealed DNAs were gel purified on a native 7% polyacrylamide gel (containing 0.5 × TBE, pre-run at 150 V for 30 min prior to loading DNA samples). DNAs were cut out of the gel, slices were crushed in TE-Low buffer (10 mM Tris pH 7.9, 0.1 mM EDTA), then nutated overnight nutation at 4 °C. DNA was isolated using Spin-X columns (Fisher Scientific) and ethanol precipitation.

### Electrophoretic mobility shift assays (EMSAs)

DNA binding reactions (20 µL) were assembled with wild type p53, p53-SNAP, or AF647-p53 (concentrations noted in figure legends) and ^32^P-DNA (0.1 nM for Fig. 1B and 0.03 nM for Figs. 1D and 3B) in 25 mM HEPES pH 7.9, 50 mM KCl, 2.5 mM MgCl_2_, 1 mM EDTA, 10% glycerol, 0.1% NP-40, 1 mM DTT and 0.05 mg/mL BSA. Reactions were incubated for 30 min at room temperature. Samples were run on 4% native polyacrylamide gels (containing 0.5 × TBE, pre-run at 150 V for 30 min) in 0.5 × TBE running buffer at 150 V for 1 h. Gels were then dried and imaged on a Typhoon 9500 Imager. Images of gels were viewed using ImageJ 1.42q software (https://imagej.nih.gov/ij/); brightness/contrast adjustments were applied equally to the entire image. Un-cropped images of the EMSAs are shown in Supplementary Fig. 7.

### Single molecule binding assays and data collection using TIRF microscopy

Cleaning and assembly of the flow chambers and preparation of stock solutions of streptavidin, d-glucose, glucose oxidase, catalase, and 100 mM Trolox (Sigma) for single molecule microscopy were as previously described^52^. All single molecule binding assays were performed at room temperature. Flow chambers were washed twice with MilliQ water and 1 × buffer (25 mM Tris 7.9, 50 mM KCl, 5 mM MgCl_2_, 10% glycerol, 0.05 mg/mL BSA, 1 mM DTT, and 0.1% NP-40) prior to use. The surface was then incubated with 0.2 mg/mL streptavidin (Sigma) and 0.8 mg/mL BSA diluted in 1 × buffer for 5 min at room temperature. Excess streptavidin was washed out with additional 1 × buffer. 10 pM of biotinylated DNA was then flowed onto the surface and incubated for 10 min to allow immobilization. Excess DNA was washed out with additional 1 × buffer. Imaging buffer (1.02 mg/mL glucose oxidase, 0.04 mg/mL catalase, and 0.83% d-glucose diluted in 1 × buffer containing 3.45 mM Trolox) was then flowed into the chamber. DNA emission movies were collected over four regions with the use of a piezo nano-positioning stage. AF647-p53 (1 nM monomer) in imaging buffer was flowed into the chamber and incubated for 10 min. Without washing, p53 + DNA emission movies were collected over the same regions as the DNA emission movies using the piezo stage.

Emission movies were collected with a 1.49 NA immersion objective based TIRF microscope (Nikon TW-2000U) equipped with two CCD cameras and a nano-positioning stage. Emission from the 635 nm laser with a power setting of 108 mW was recorded by a Cascade II Photometric CCD camera using the NIS-elements software. Emission movies of DNA only were collected with a 60 ms frame rate for 100 frames over 4 separate regions (512 × 512-pixels, 200 nm per pixel) per slide. Using the nano-positioning stage, emission movies of p53 + DNA were then collected over the same 4 regions with a 60 ms frame rate for 1000 frames to obtain bound dwell times for the wild type and mutant DNAs. To obtain unbound dwell times on wild type DNA the data were collected with a 200 ms frame rate for 2000 frames, and a 600 ms frame rate for 1000 frames; for the mutant half site DNA these parameters were 60 ms for 5000 frames.

### Analysis of single molecule data

In-house software written in IDL was used to co-localize spots in the DNA and p53 + DNA movies, quantify the oligomeric state of bound p53, and extract dwell times. Briefly, for co-localization of the fluorescent DNA and AF647-p53 spots, a gaussian of spot density was built for the entire movie. Spots near the edges of the image were not used in analysis; typically a region of ~ 480 × 400 pixels was analyzed, with a total of ~ 750 DNA spots. Supplementary Fig. 1 shows images of slide surfaces with DNA alone, DNA + AF647-p53, and AF647-p53 alone. To computationally identify potential spot pairs a pixel-by-pixel sum was performed for the frames of a DNA movie and separately for the frames of a paired DNA + AF647-p53 movie of the same slide region. The locations of fluorescent spots were identified in each of the summed images by searching an image for a peak of intensity that fell within user input intensity thresholds, which once set, remained consistent for the entire analysis. The locations of spots in the two movies were compared and any spot between the movies within 2 pixels of one another were considered a potential spot pair. After computationally finding potential spot pairs, each pair was manually visualized and evaluated, and those with anomalies were rejected. Spot pairs were rejected if, for example, the Gaussian for an individual spot showed multiple peaks, indicating that two spots were likely too close together to distinguish which was used for colocalization.

For a spot pair found between the DNA movie and p53 + DNA emission movie, the intensity trace from the DNA emission movie was used to calculate a base red intensity (N) that corresponded to the value of a single AF647 dye. The value N was used to calculate the number of dyes present for each state in the p53 + DNA intensity trace of the spot pair. The total number of dyes was used to characterize a state as being unbound (one dye, N) or bound by tetrameric p53 (four or five total dyes; 4 N or 5 N). Transitions between different intensity states representing dissociation or association of p53 tetramers were identified. Potential state changes were computationally distinguished from noise by calculating the noise and standard deviation of the noise within the data in each time trace, and comparing frame-by-frame changes in intensity to the noise. An increase or decrease was computationally considered a potential state change if the change in intensity between adjacent frames was greater than two times the noise, greater than three times the standard deviation in the noise, and at least a 20% change. All computationally identified state changes were further considered manually and those with anomalies were rejected. The number of unbound DNA states, dynamic tetrameric p53/DNA bound states, bound dwell times, and unbound dwell times between states were calculated then compiled for all regions for a given experiment. Spots showing bound p53 with no transitions across the time of collection were not included in subsequent analyses. To determine rate constants, dwell times from independent replicate experiments were combined and plotted as cumulative sums. The data were initially fit with a single exponential equation using Prism 8 software (https://www.graphpad.com) to obtain rate constants and 95% confidence intervals of the fit. In the cases of bound dwell times measured with the wild type and 5 bp insert DNAs, the data were not well fit with a single exponential equation, hence a double exponential equation was used. We reported the value of each rate constant (k) with the smallest number of significant digits that showed a meaningful range for the value of k in the 95% confidence interval. The half times for binding and unbinding were calculated from the equation for the half-time of a first-order reaction: t_1/2_ = ln2/k. The value for tau in Table 1 is 1/k.

## Supplementary information

Supplementary Information.

## Data Availability

The raw single molecule data and IDL analysis software used in this study are publicly available on the Open Science Framework platform. The raw gel images are in Supplementary Fig. 7.
