# Supplementary material for: Single molecule studies reveal that p53 tetramers dynamically bind response elements containing one or two half sites
Source: Sci Rep. 2020 Sep 30;10:16176. doi: 10.1038/s41598-020-73234-6 (PMC7528078; doi:10.1038/s41598-020-73234-6)
Supplement: Supplementary file 1 — Supplementary Information. [file 41598_2020_73234_MOESM1_ESM.pdf]

Supplementary Information

Single molecule studies reveal that p53 tetramers dynamically bind response elements containing one or two half sites

Elina Ly, Jennifer F. Kugel, James A. Goodrich

Supplementary Figures 1-7

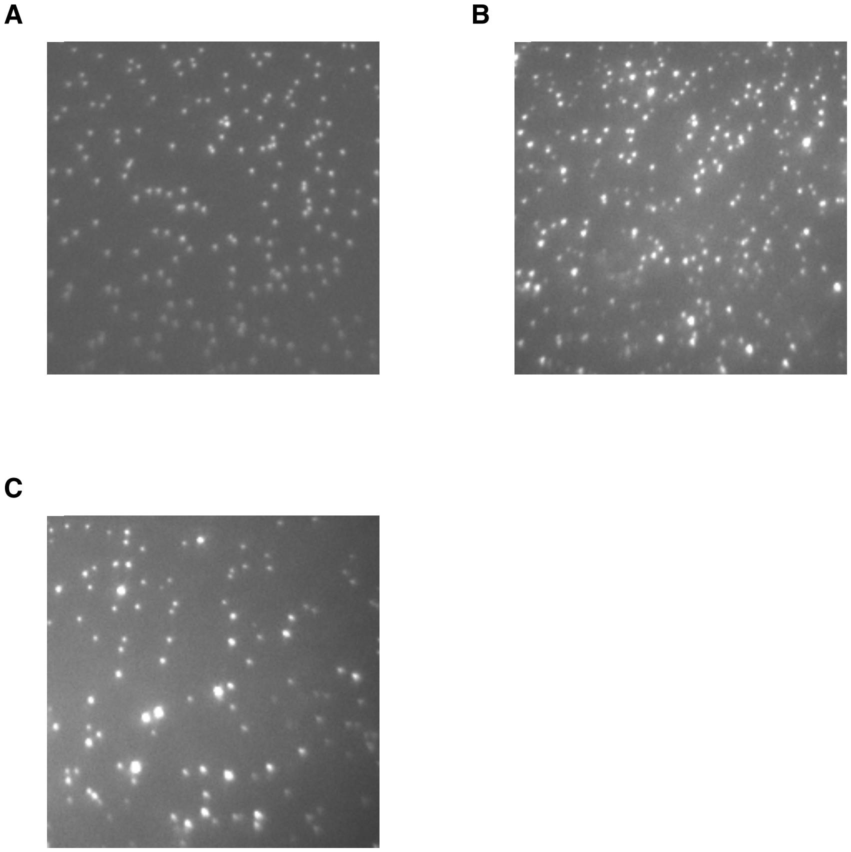

Supplementary Figure 1. Representative images of slide surfaces. (A) Shown is a 200 x 200 pixel region of a slide surface containing immobilized DNA labeled with AF647. The image is a pixel-per-pixel sum of 100 frames of red emission data. **(B)** Shown is the same region as panel A, after flowing in AF647-p53. **(C)** Shown is a 200 x 200 pixel region of a slide with no DNA, imaged after flowing in AF647-p53.

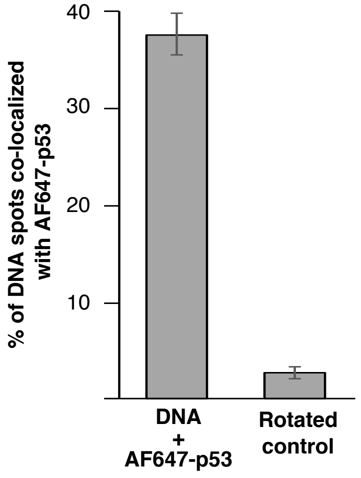

Supplementary Figure 2. Single molecule fluorescence co-localization allows us to specifically detect binding of p53 to immobilized DNA. Plotted is the percentage of DNA molecules with a co-localized AF647-p53 from an experiment with wild type DNA (left bar). When the AF647-p53 image was rotated 90° (right bar) the percentage of DNA with a co-localized AF647-p53 dropped dramatically. Each bar is the average of 4 regions and the errors are the standard deviations.

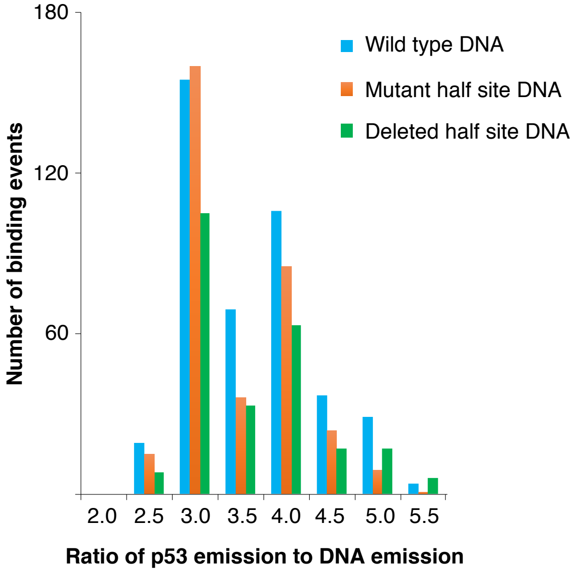

Supplementary Figure 3. Tetrameric binding events show three or four active AF647 dyes. For each binding event that our software identified as tetrameric, we subtracted the DNA only emission from p53+DNA emission to obtain the bound p53 emission. We then calculated the ratio of bound p53 emission to DNA emission and plotted the data in a histogram to visualize the distribution. For each DNA, the data are consistent with tetrameric binding events showing three or four active AF647 dyes. The wild type DNA includes 419 binding events, the mutant DNA 330 events, and the deleted half site DNA 249 events.

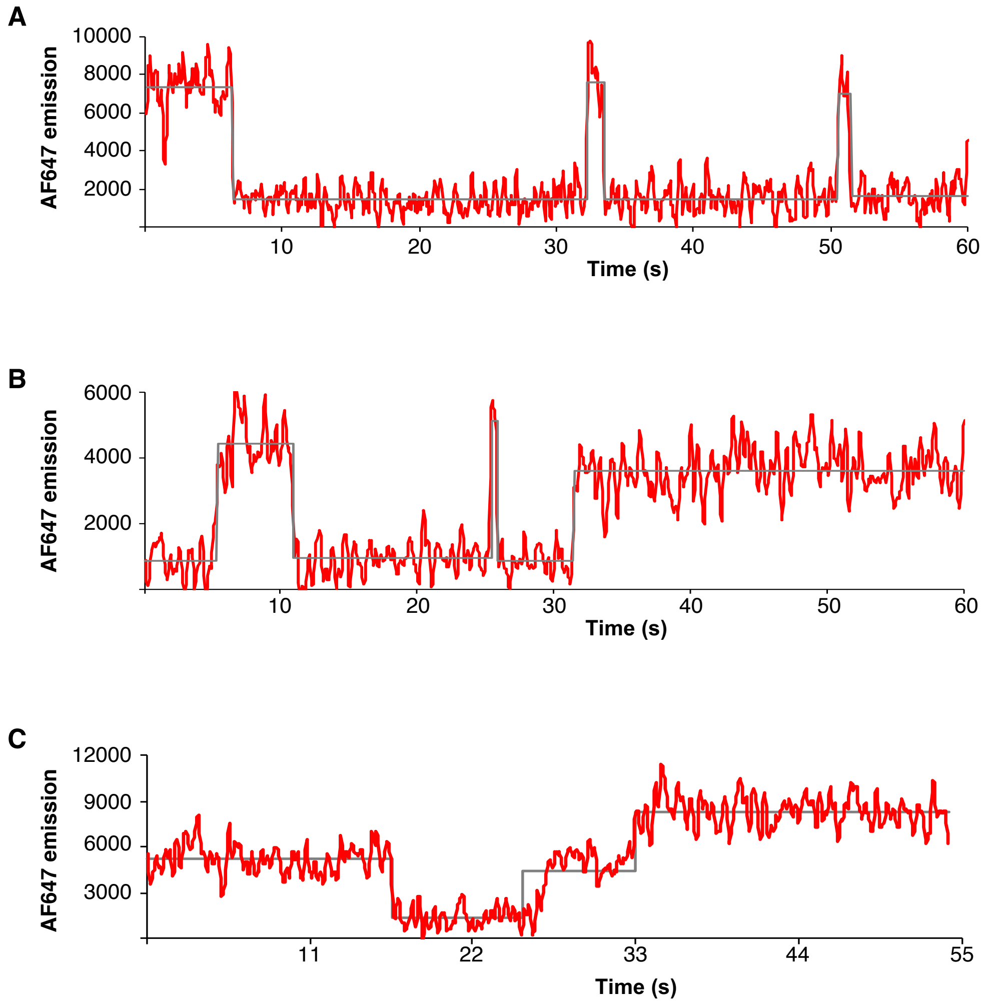

Supplementary Figure 4. Representative AF647 emission data from single DNA molecules with p53 binding events. (A)-(B) Shown are tetrameric binding events on single DNAs. The red line shows AF647 emission and the gray line shows state transitions between free DNA and tetramer/DNA complexes. **(C)** Shown is an example of a rare time trace that demonstrates a binding event containing a dimer to tetramer transition observed on a single DNA molecule. Note that these events are in the vast minority, only representing 1-2% of total state changes observed. The red line shows AF647 emission and the gray line shows state transitions between free DNA, dimer/DNA, and tetramer/DNA complexes.

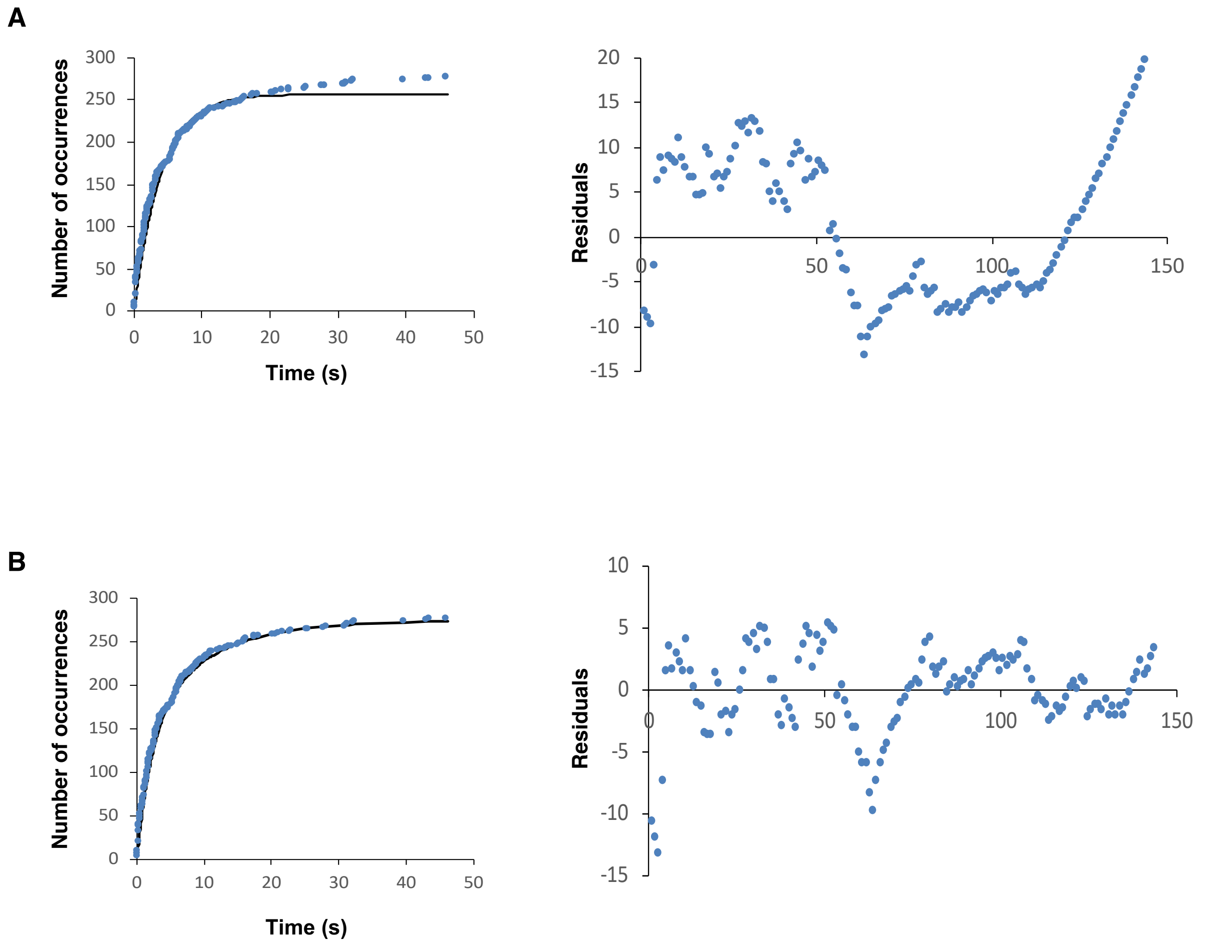

Supplementary Figure 5. Plots of residuals from the single and double exponential fits of the bound dwell times on wild type DNA. (A) The plot on the left is the cumulative sums of the bound dwell times obtained from two replicate experiments for tetrameric p53 binding wild type DNA. The data were fit with a signal exponential equation. The residuals are plotted on the right and clearly reflect that the data were systematically above the curve at the longer dwell times. **(B)** The plot on the left is the same data as in panel A, fit with a double exponential equation. The residuals are plotted on the right and clearly reflect that this equation better fits the data.

A

B

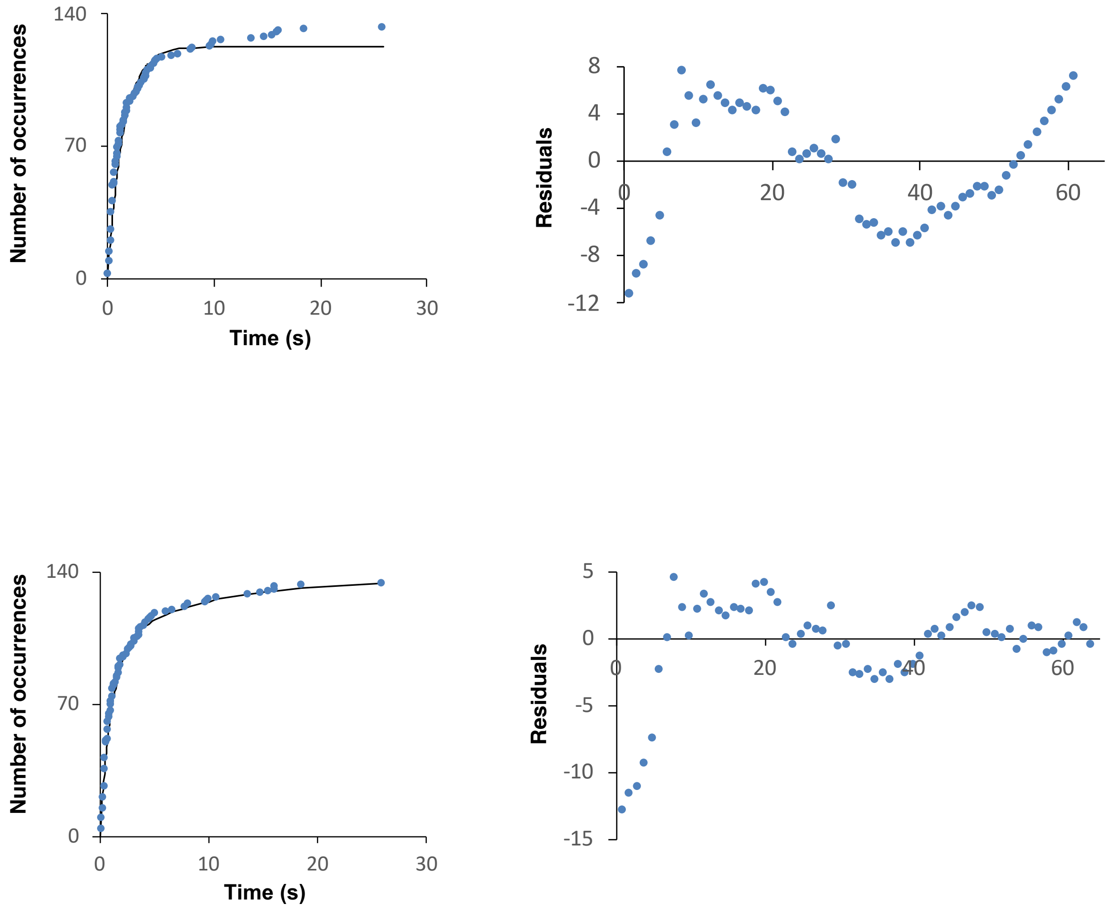

Supplementary Figure 6. Plots of residuals from the single and double exponential fits of the bound dwell times on the +5bp insert DNA. (A) The plot on the left is the cumulative sums of the bound dwell times obtained from two replicate experiments for tetrameric p53 binding +5bp insert DNA. The data were fit with a signal exponential equation. The residuals are plotted on the right and clearly reflect that the data were systematically above the curve at the longer dwell times. **(B)** The plot on the left is the same data as in panel A, fit with a double exponential equation. The residuals are plotted on the right and clearly reflect that this equation better fits the data.

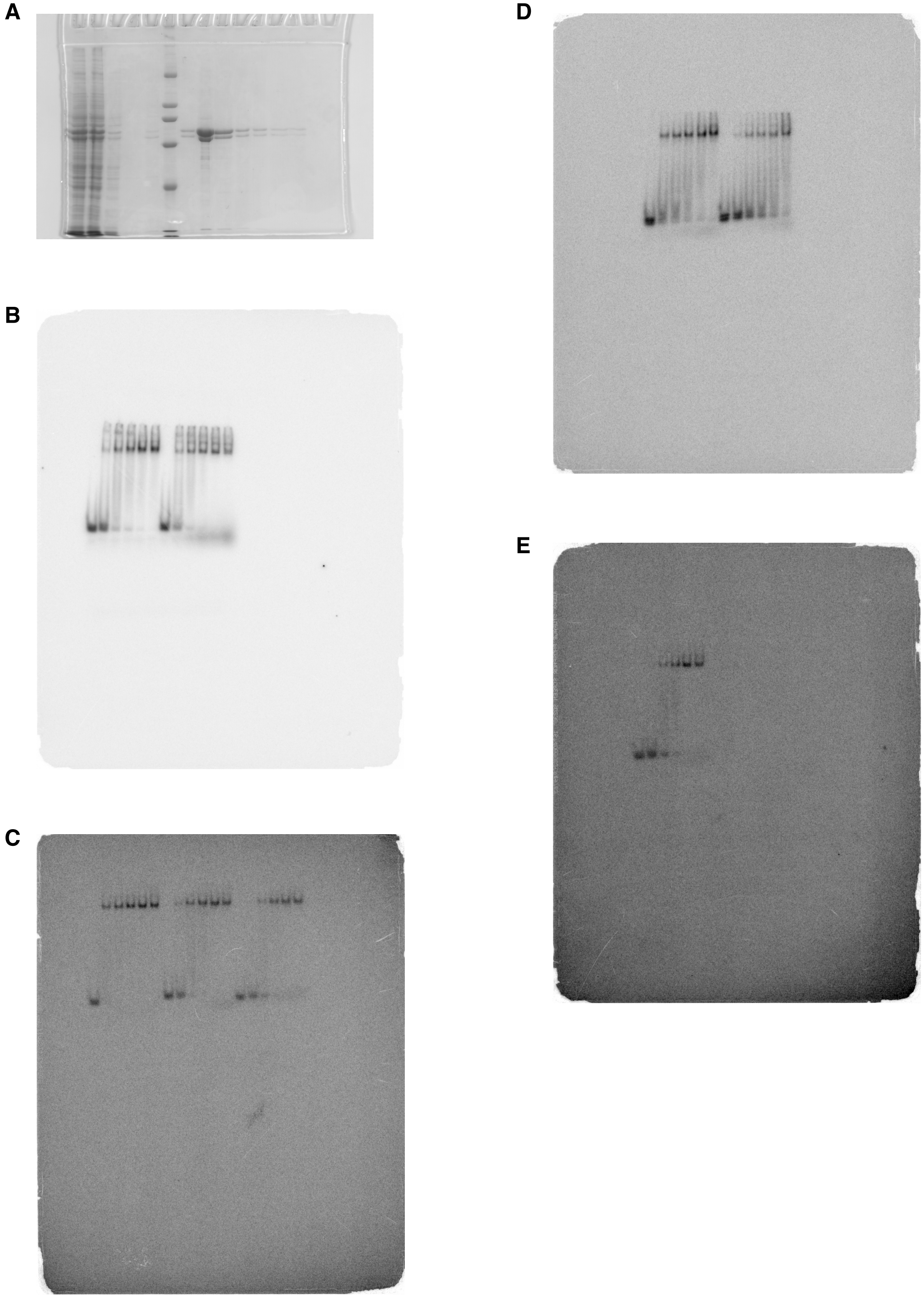

Supplementary Figure 7. Uncropped images of gels. (A) Data for Figure 1A. **(B)** Data for Figure 1B, wild type p53 is in the first 6 lanes of gel. **(C)** Data for Figure 1D. **(D)** Data for Figure 1B p53-SNAP is in the first 6 lanes of gel. **(E)** Data for Figure 3B.
